# Revisiting the Enhanced Red Upconversion Emission from a Single β-NaYF_4_:Yb/Er Microcrystal By Doping with Mn^2+^ Ions

**DOI:** 10.1186/s11671-019-2931-0

**Published:** 2019-03-19

**Authors:** Maohui Yuan, Rui Wang, Chaofan Zhang, Zining Yang, Xu Yang, Kai Han, Jingfeng Ye, Hongyan Wang, Xiaojun Xu

**Affiliations:** 1 College of Advanced Interdisciplinary Studies, National University of Defense Technology, Changsha, 410073 China; 2 State Key Laboratory of Pulsed Power Laser Technology, National University of Defense Technology, Changsha, 410073 China; 3 Hunan Provincial Key Laboratory of High Energy Laser Technology, National University of Defense Technology, Changsha, 410073 China; 4 State Key Laboratory of Laser Interaction with Matter, Northwest Institute of Nuclear Technology, Xi’an, 710024 China

**Keywords:** Upconversion emission, Multicolor display, Single β-NaYF_4_ microcrystal, Manganese ions (Mn^2+^), Energy transfer

## Abstract

The presence of manganese ions (Mn^2+^) in Yb/Er-co-doped nanomaterials results in suppressing green (545 nm) and enhancing red (650 nm) upconversion (UC) emission, which can achieve single-red-band emission to enable applications in bioimaging and drug delivery. Here, we revisit the tunable multicolor UC emission in a single Mn^2+^-doped β-NaYF_4_:Yb/Er microcrystal which is synthesized by a simple one-pot hydrothermal method. Excited by a 980 nm continuous wave (CW) laser, the color of the single β-NaYF_4_:Yb/Er/Mn microrod can be tuned from green to red as the doping Mn^2+^ ions increase from 0 to 30 mol%. Notably, under a relatively high excitation intensity, a newly emerged emission band at 560 nm (^2^H_9/2_ → ^4^I_13/2_) becomes significant and further exceeds the traditional green (545 nm) emission. Therefore, the red-to-green (R/G) emission intensity ratio is subdivided into traditional (650 to 545 nm) and new (650 to 560 nm) R/G ones. As the doped Mn^2+^ ions increase, these two R/G ratios are in lockstep with the same tunable trends at low excitation intensity, but the tunable regions become different at high excitation intensity. Moreover, we demonstrate that the energy transfer (ET) between Mn^2+^ and Er^3+^ contributes to the adjustment of R/G ratio and leads to tunable multicolor of the single microrod. The spectroscopic properties and tunable color from the single microrod can be potentially utilized in color display and micro-optoelectronic devices.

## Introduction

Photon UC in lanthanide-doped nanomaterials has drawn much attention recently due to their superior spectroscopic properties [1, 2]. As the most significant near-infrared (NIR) to visible UC structures, the lanthanide-sensitized UC based on the ET from Yb^3+^ to Er^3+^(Tm^3+^/Ho^3+^) in β-NaYF_4_ nanocrystals has been studied intensively owing to its promising applications in color display [3, 4], super-resolution nanoscopy [5, 6], security printing [7, 8], laser materials [9–11], and biological luminescent labels [12–14]. It is well-known that lanthanide ions have an abundance of 4f^N^ electronic states, which typically generate multi-band emissions [15]. However, multi-band emissions preclude the quantitative imaging of samples targeted with multiple upconverting probes and reduce the sensitivity of imaging [16]. Therefore, some efforts have been made to achieve single-band UC emissions [17–19]. For example, the introduction of transition metals (Mn^2+^, etc.) into Yb/Er co-doped nanomaterials can enhance the R/G (650-to-545 nm) ratio and achieve single-red-band emission because of the strongly ET process between Er^3+^ and Mn^2+^ [20–24].

To date, some studies have been performed to investigate the Mn^2+^-doped Yb/Er nanocrystals for applications in bioimaging [20, 25], sensor [26–28], and biomarker detection [29]. In fact, compared to the nanocrystals, microcrystals fascinate more advantages for applications in micro-optoelectronic devices [30], volumetric color display [31, 32], and microlasers [11] based on their high crystallinity and luminescent efficiency [33]. Consequently, it is very important for us to study the UC luminescence properties of the microcrystals. However, most present measurements of the samples were executed by powders or in organic solvents, which can cause severe overheating problems and be influenced by adjacent microcrystals [34]. Thus, exploring the UC luminescence and tunable color from the single microcrystal can effectively avoid the influence of the environment and broaden its further applications in micro-optoelectronic devices.

Moreover, excited by 980 nm CW laser, Yb/Er co-doped materials usually emit red (650 nm) and green (525 and 545 nm) UC emissions, as well as a weaker blue (410 nm) emission. Generally, the red and green UC emissions dominate the spectra, and the blue emission remains relatively weak. In comparison with these three UC emissions, other UC emissions are rarely observed in Yb/Er co-doped materials. Previously, we have observed a newly emerged 560 nm (^2^H_9/2_ → ^4^I_13/2_) emission from a single β-NaYF_4_:Yb/Er microcrystal under saturated excitation [35]. As excitation intensity increases, the 560-nm emission increases rapidly and further exceeds the traditional green emission (545 nm). However, for Yb/Er/Mn tri-doped materials, the transition of ^2^H_9/2_ → ^4^I_13/2_ (560 nm) in Er^3+^ also acts as an ET channel populating the level ^4^T_1_ of Mn^2+^, which, to the best of our knowledge, has not been reported or explored so far. Thus, for Yb/Er/Mn tri-doped materials, the suppressing of the new green (560 nm) UC emission and the tuning of R/G ratio remain largely unknown. Therefore, as discussed above, exploiting the UC emissions from single β-NaYF_4_:Yb/Er/Mn microcrystal can help us understand the tuning of new R/G ratio and expand its scope of applications in micro-optoelectronic devices.

In this work, we have synthesized Mn^2+^-doped β-NaYF_4_:Yb/Er microcrystals through a simple one-pot hydrothermal method. The UC emission properties and the relevant luminescence color from a single microcrystal were investigated using a high-performance luminescence collection system including an inverted fluorescence microscope with a × 100 objective lens (NA = 1.4). Excited by a 980-nm CW laser, the luminescence color can be tuned from green to red when gradually increasing the doped Mn^2+^ ions from 0 to 30 mol%. The tuning R/G ratios for traditional 650 to 545 nm and new 650 to 560 nm have been discussed in detail. The mechanism of the tunable UC emission color was also demonstrated based on the ET process between Mn^2+^ and Er^3+^.

## Methods

### Materials

The raw materials were purchased from Aladdin (China): Y_2_O_3_ (99.99% metals basis), Yb_2_O_3_ (99.99% metals basis), Er_2_O_3_ (99.99% metals basis), MnCl_2_·4H_2_O (99% metals basis), nitric acid (HNO_3_, analytical reagent), ethylenediaminetetraacetic acid disodium salt dihydrate (EDTA-2Na, analytical reagent), sodium hydroxide (NaOH, analytical reagent), ammonium fluoride (NH_4_F, analytical reagent). All the chemicals were used as received directly without further purification.

### Synthesis of β-NaYF_4_ Microcrystals

We synthesized the β-NaYF_4_:Yb/Er/Mn (20/2/× mol%) microcrystals through a modified hydrothermal method. The Y_2_O_3_, Yb_2_O_3_, and Er_2_O_3_ powders were dissolved in dilute nitrate solution and heated to remove the residual nitrate, yielding a clear solution of Ln (NO_3_)_3_ (0.2 M). In a typical procedure, the EDTA-2Na (1 mmol) and NaOH (6 mmol) were mixed with 13.5 mL deionized (DI) water under continuously stirring in a flask yielding a clear solution. Then, 5 mL of MnCl_2_ (0.2 M) and Ln (NO_3_)_3_ (0.2 M) aqueous solutions, 8 mL of NH_4_F (2.0 M) aqueous solutions, and 7 mL of dilute hydrochloric acid (1 M) were injected into the flask. The mixtures were stirred for 1.5 h and then transferred into a 50 mL Teflon-lined autoclave and heated at 200 °C for 40 h. The as-obtained precipitates were collected by centrifugation, washed with DI water and ethanol for several times, and finally dried in air at 40 °C for 12 h. Microcrystals with different concentrations of Mn^2+^ can be achieved by varying the volume of MnCl_2_ aqueous solutions (the total Y^3+^, Yb^3+^, Er^3+^, and Mn^2+^ ions content were kept constant at 1 mmol).

### Physical Characterization

X-ray diffraction (XRD) patterns of the microcrystals were measured using X-ray diffractometer with Cu K radiation at 40 kV and 200 mA (Rigaku). The morphology of the β-NaYF_4_:Yb/Er/ (20/2/× mol%) microcrystals was characterized by scanning electron microscope (SEM) (S4800, Hitachi).

### Photoluminescence Measurements

For photoluminescence experiments, the 980-nm CW laser was introduced into an inverted microscope (Observer A1, Zeiss) and focused on the microcrystals by using a × 100 objective lens (NA = 1.4). The diameter of the excitation spot was estimated to be ~ 2.0 μm. The UC luminescence was collected by the same objective lens and then delivered to a spectrometer (SR-500I-B1, Andor) equipped with a charge-coupled device (CCD) (DU970N, Andor) for analysis. The luminescence color of the single microcrystal was recorded by using a camera (DS-Ri2, Nikon). The UC luminescence lifetime was measured by using a digital oscilloscope (1 GHz, InfiniiVsionDSOX6002A, KEYSIGHT) and a nanosecond pulsed laser (with a pulse duration of 20 ns and a repetition rate of 10 Hz) as the excitation source. All the measurements were performed at room temperature.

## Results and Discussion

**Fig. 1:**
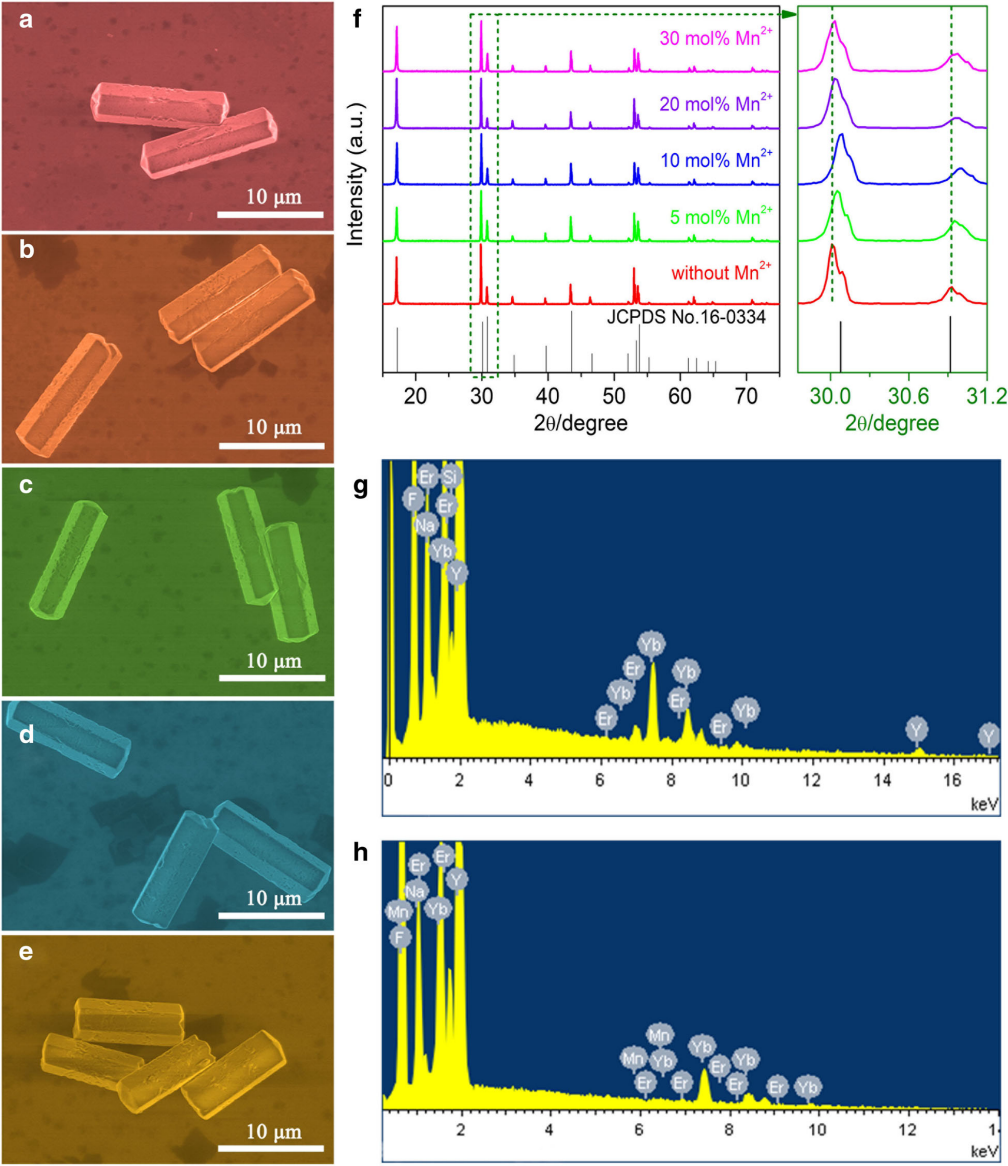
SEM micrographs of the NaYF_4_:Yb/Er (20/2 mol%) microcrystals doping with **a** 0, **b** 5, **c** 10, **d** 20 and **e** 30 mol% Mn^2+^ ions, respectively. **f** XRD patterns of the NaYF_4_ microcrystals doping with different concentrations of Mn^2+^ ions. EDS analysis of the NaYF_4_:Yb/Er (20/2 mol%) microcrystals doping with **g** 0 and **h** 30 mol% Mn^2+^ ions, respectively

The typical morphologies of the β-NaYF_4_:Yb/Er/Mn (20/2/x mol%) microcrystals are characterized by SEM images, as shown in Fig. 1a–e. It indicates that the microcrystals exhibit a pure hexagonal phase of morphology and uniform size with the diameters of ~ 3.5 μm and lengths of ~ 13 μm. Notably, the length of the microcrystals slightly reduces to 10 μm as the doping Mn^2+^ ions increase to 30 mol%. Figure 1g shows the XRD patterns of the β-NaYF_4_:Yb/Er microcrystals doped with different concentrations of Mn^2+^ ions. All the diffraction peaks were in good agreement with the standard hexagonal phase of NaYF_4_ (JCPDS No. 16-0334). Importantly, as the doping Mn^2+^ ions increase, the microcrystals still maintain a hexagonal phase and no other impurity peaks are observed. It reveals that the addition of Mn^2+^ ions has no influence on the morphology and crystal phase of the β-NaYF_4_ microcrystals. Furthermore, some diffraction peaks firstly shift slightly towards to higher angles as the doping Mn^2+^ ions gradually increase from 0 to 10 mol%, and then drift back to lower angles when further increasing the Mn^2+^ ions up to 30 mol%. The results probably indicate that the smaller Mn^2+^ ions (*r* = 1.10 Å) mainly occupy the larger Na^+^ (*r* = 1.24 Å) sites at low Mn^2+^ concentrations (less than 10 mol%), and then insert the Y^3+^ (*r* = 1.159 Å) sites in NaYF_4_ host lattice with the Mn^2+^ ions increasing up to 30 mol% [4, 36, 37]. We have also performed the compositional analysis on the β-NaYF_4_ microcrystals by EDS, as shown in Fig. 1g–h. The EDS analysis confirms the presence of Na, F, Y, Yb, and Er elements in Mn-free β-NaYF_4_:Yb/Er microcrystals (Fig. 1g). In comparison, the Mn element is found in β-NaYF_4_:Yb/Er microcrystals doping with 30 mol% Mn^2+^ ions (Fig. 1h), indicating the Mn^2+^ ions are well embedded in the NaYF_4_ host lattice.

**Fig. 2:**
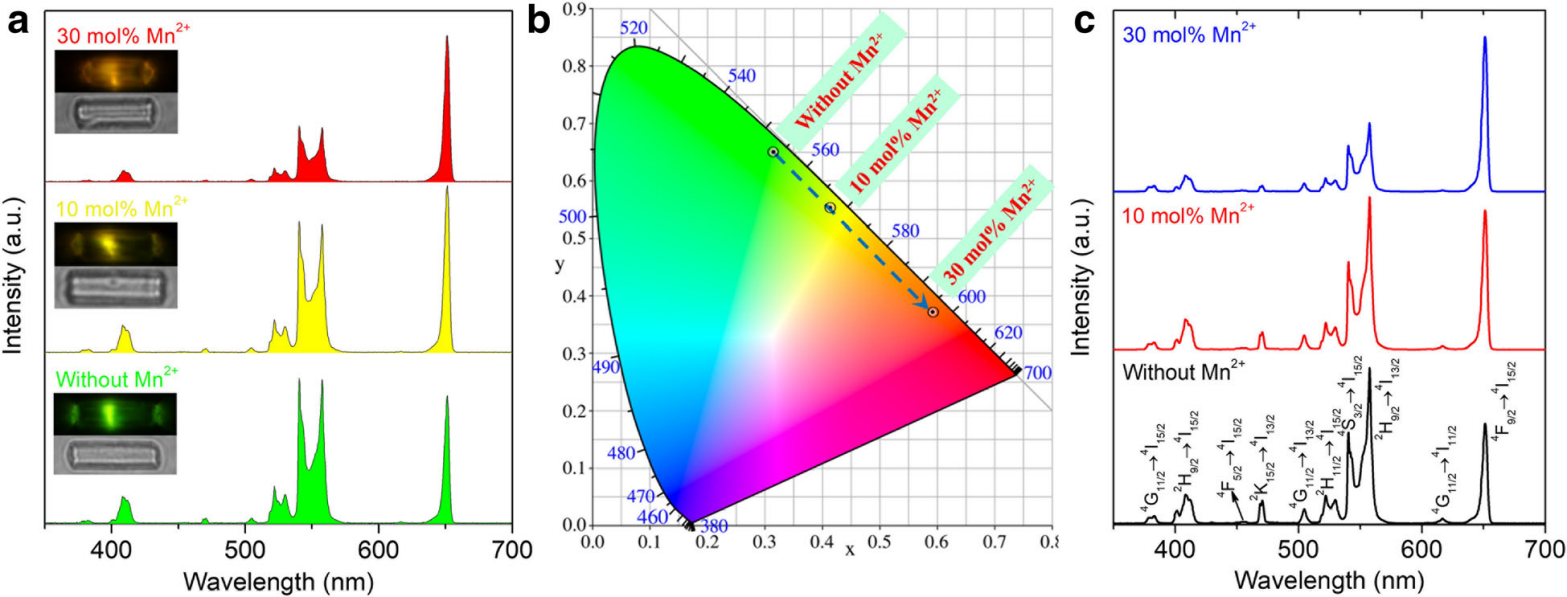
**a** UC emission spectra from a single β-NaYF_4_:Yb/Er (20/2 mol%) microcrystal doping with 0, 10, and 30 mol% Mn^2+^ ions under the excitation intensity of 1.59 kW cm^−2^. **b** CIE chromaticity coordinates for the UC luminescence of the single β-NaYF_4_:Yb/Er (20/2 mol%) microcrystals doping with different amounts of Mn^2+^ ions. **c** UC emission spectra from a single β-NaYF_4_:Yb/Er (20/2 mol%) microcrystal doping with 0, 10, and 30 mol% Mn^2+^ ions under the excitation intensity of 95.52 kW cm^−2^

Figure 2a shows the UC emissions from a single β-NaYF_4_:Yb/Er (20/2 mol%) microcrystal doped with different amounts of Mn^2+^ ions under a relatively low excitation intensity (1.59 kW cm^−2^). The insets show the single microcrystal and its corresponding luminescence color observed from the microscope. Three major emission bands are indexed in the spectra, which are ascribed to the transitions of ^2^H_9/2_ → ^4^I_15/2_ (410 nm), (^2^H_11/2_/^4^S_3/2_) → ^4^I_15/2_ (525 and 545 nm), and ^4^F_9/2_ → ^4^I_15/2_ (650 nm) from Er^3+^, respectively. For the Mn-free single microcrystal, the green (545 nm) emission dominates the emission spectrum, leading to the single microcrystal lighting up with a green luminescence color. With increasing the doping Mn^2+^ ions, the red (650 nm) emission grows remarkably and gradually exceeds the green emission and dominates the spectrum in the end as the dopant Mn^2+^ ions reach 30 mol%. Thus, the luminescence color can be tuned from green to yellow and finally becomes red. Figure 2b displays the calculated CIE chromaticity coordinates based on the UC emission spectrums in Fig. 2a. It is obvious that the UC luminescence color changes from green to red with the doping Mn^2+^ ions increasing from 0 to 30 mol%. As shown in Fig. 2c, it is interesting to observe several new UC emission bands as the excitation intensity increases up to 95.52 kW cm^−2^. These new UC emissions can be detected both in Mn-free and highly Mn^2+^-doped microcrystals. As demonstrated in our previous study [35], these new UC emissions originate from the transitions of ^4^G_11/2_ → ^4^I_15/2_ (382 nm), ^4^F_5/2_ → ^4^I_15/2_ (457 nm), ^2^K_15/2_ → ^4^I_13/2_ (472 nm), ^4^G_11/2_ → ^4^I_15/2_ (506 nm), ^2^H_9/2_ → ^4^I_13/2_ (560 nm), and ^4^G_11/2_ → ^4^I_11/2_ (618 nm) in Er^3+^, respectively. It is noteworthy that the newly emerged UC emissions can be observed regardless of the Mn^2+^ concentration and the new 560 nm emission is always stronger than the traditional green (545 nm) emission.

**Fig. 3:**
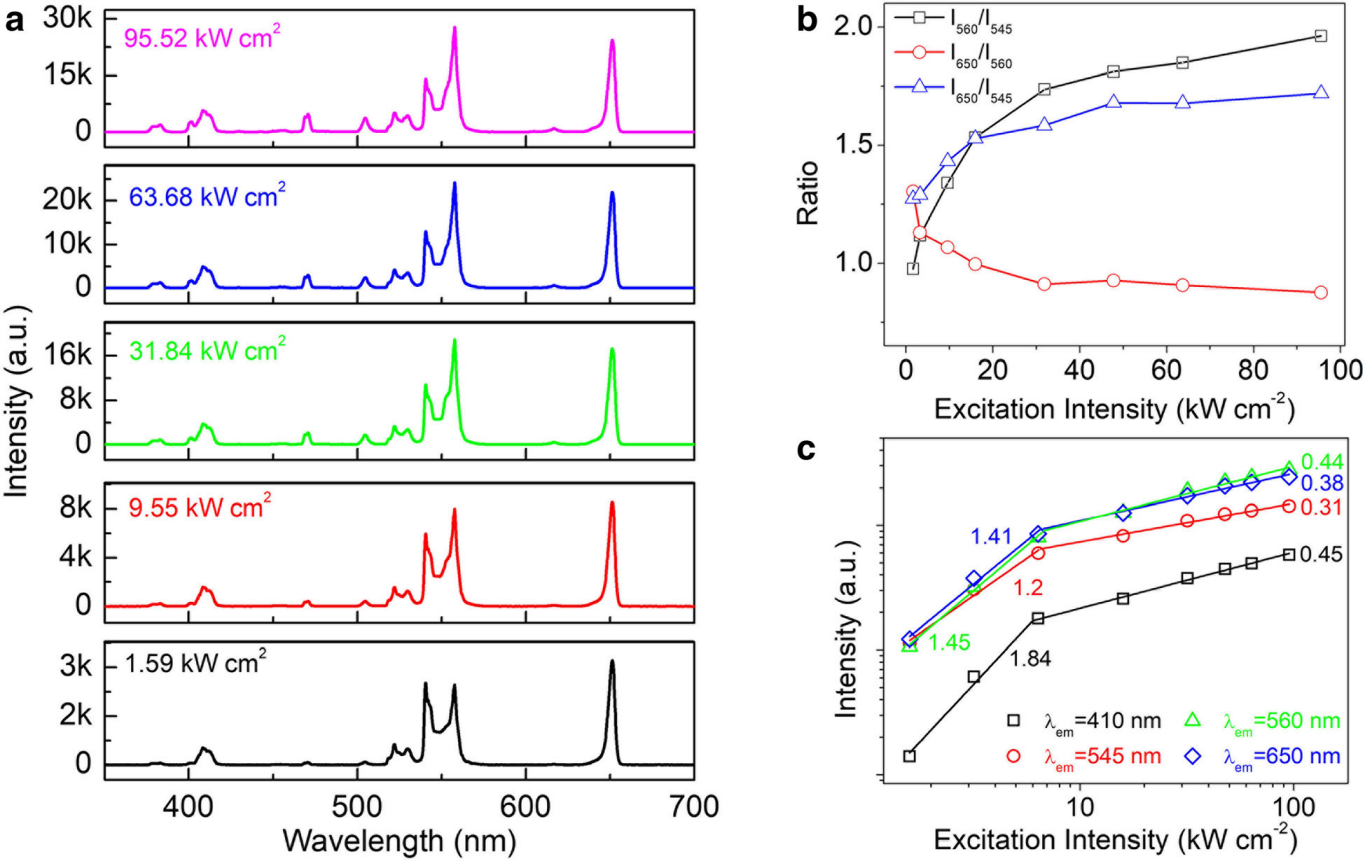
**a** UC emission spectra from a single β-NaYF_4_:Yb/Er/Mn (20/2/10 mol%) microcrystal irradiated with different excitation intensities. **b** The UC emission intensity ratios for a single β-NaYF_4_:Yb/Er/Mn (20/2/10 mol%) microcrystal as a function of the excitation intensities. **c** The dependence of the UC emission intensity on the excitation intensity for a single β-NaYF_4_:Yb/Er/Mn (20/2/10 mol%) microcrystal

To clearly identify these new UC emissions, we demonstrated the UC emissions from the single β-NaYF_4_:Yb/Er/Mn (20/2/10 mol%) microcrystal under different excitation intensities, as shown in Fig. 3a. At the excitation intensity of 1.59 kW cm^−2^, the red emission (650 nm) is much stronger than the traditional green emission (545 nm), and the new 560 nm UC emission is lower than the traditional green (545 nm) emission. Furthermore, the UC emissions centered at 382, 506, and 472 nm can be distinguished from the spectra. When increasing the excitation intensity up to 9.55 kW cm^−2^, the 560-nm emission exceeds the 545 nm and becomes comparable to the red emission (650 nm). Moreover, the 506- and 472-nm emissions become more efficient. If we further increase the excitation intensity up to 31.84 kW cm^−2^, the 560-nm emission increases dramatically and exceeds the traditional red emission (650 nm). This is different from the previous reports in which doping the Mn^2+^ ions only facilitated the enhancement red emission and in which the new 560-nm UC emission was not observed. Meanwhile, the newly emerging emission bands at 382, 506, and 472 nm further increase with the excitation intensity rising to 95.52 kW cm^−2^. In Fig. 3b, we calculated the R/G ratios for a single β-NaYF_4_:Yb/Er/Mn (20/2/10 mol%) microcrystal under different excitation intensities. The ratio (560 to 545 nm) increases from ~ 0.97 to 1.96 with the excitation intensities varying from 1.59 to 95.52 kW cm^2^. However, the traditional R/G ratio (650 to 545 nm) rises from 1.27 to 1.72 and the new R/G ratio (650 to 560 nm) decreases from 1.31 to 0.87 as the excitation intensities increase. Figure 3c demonstrates the dependence of the UC emission intensity on the excitation intensity for the single β-NaYF_4_:Yb/Er/Mn (20/2/10 mol%) microcrystal. Under low power excitation, the slopes of the four UC emissions are all close to ~ 2. Moreover, these slopes become less than 1 under high power excitation, which should be attributed to the saturation effect [38–41].

**Fig. 4:**
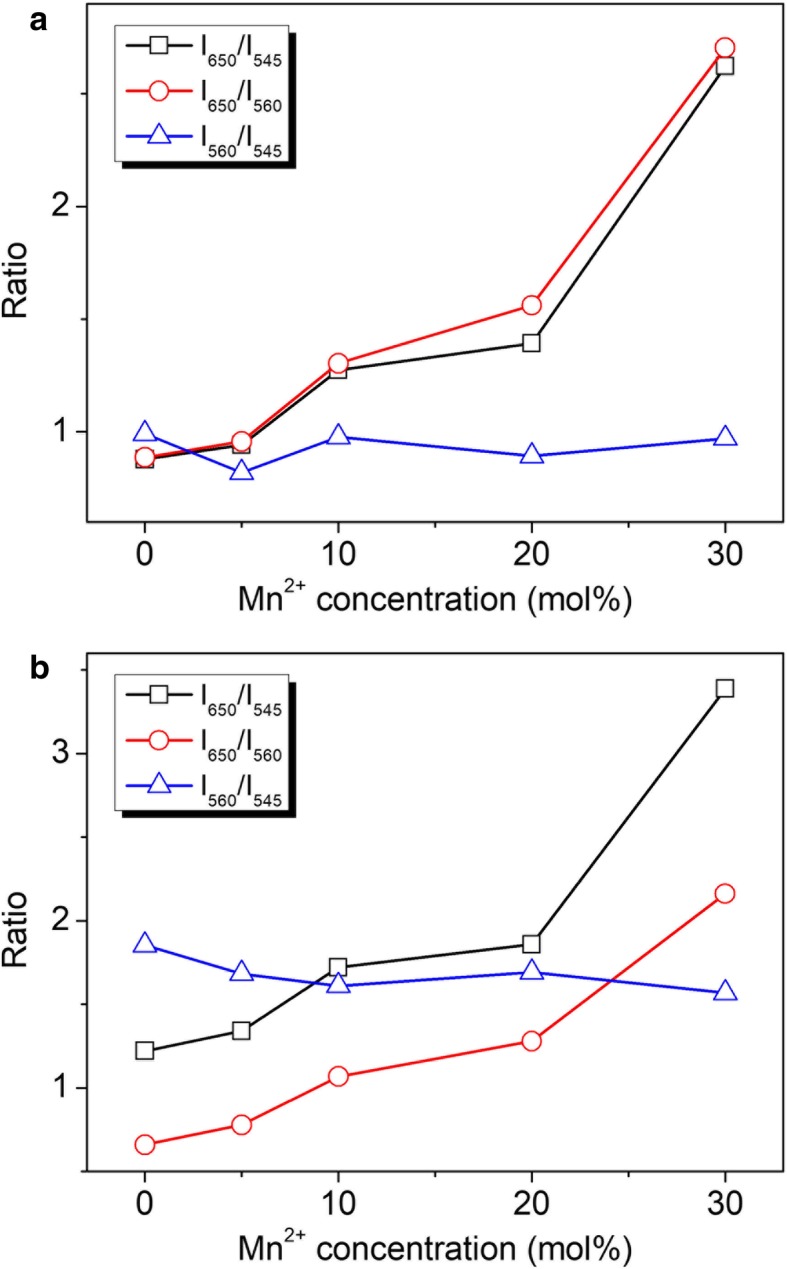
The UC emission intensity ratios for a single β-NaYF_4_:Yb/Er (20/2 mol%) microcrystal doping with different concentrations of Mn^2+^ ions under the excitation intensity of (**a**) 1.59 kW cm^−2^ and (**b**) 95.52 kW cm^−2^

Figure 4 shows the tunable UC emission intensity ratios for the single β-NaYF_4_:Yb/Er (20/2 mol%) microcrystal doping with different concentrations of Mn^2+^ under low and high excitation intensity. The ratio (560 to 545 nm) is less than 1 under low power excitation but becomes larger than 1.5 at high power excitation. At low power excitation (Fig. 4a), the traditional R/G (650 to 545 nm) ratio is basically consistent with the new R/G (650 to 560 nm) ratio. These two ratios (650 to 545 and 650 to 560 nm) start from ~ 0.87 and then gradually increase to ~ 2.7 with the doping Mn^2+^ ions varying from 0 to 30 mol%. Nevertheless, these two ratios become different under high excitation intensity (Fig. 4b). The traditional ratio (650 to 545 nm) rises from ~ 1.2 to 3.4, whereas the new ratio (650 to 560 nm) increases from 0.66 to 2.15 when the doped Mn^2+^ ions growing from 0 to 30 mol%. It reveals that the traditional and new R/G ratios exhibit different tunable trends under low and high excitation intensity. The newly emerged 560-nm UC emission changes the tunability of multicolor UC emissions, which is different from the previously reported results [20–24].

**Fig. 5:**
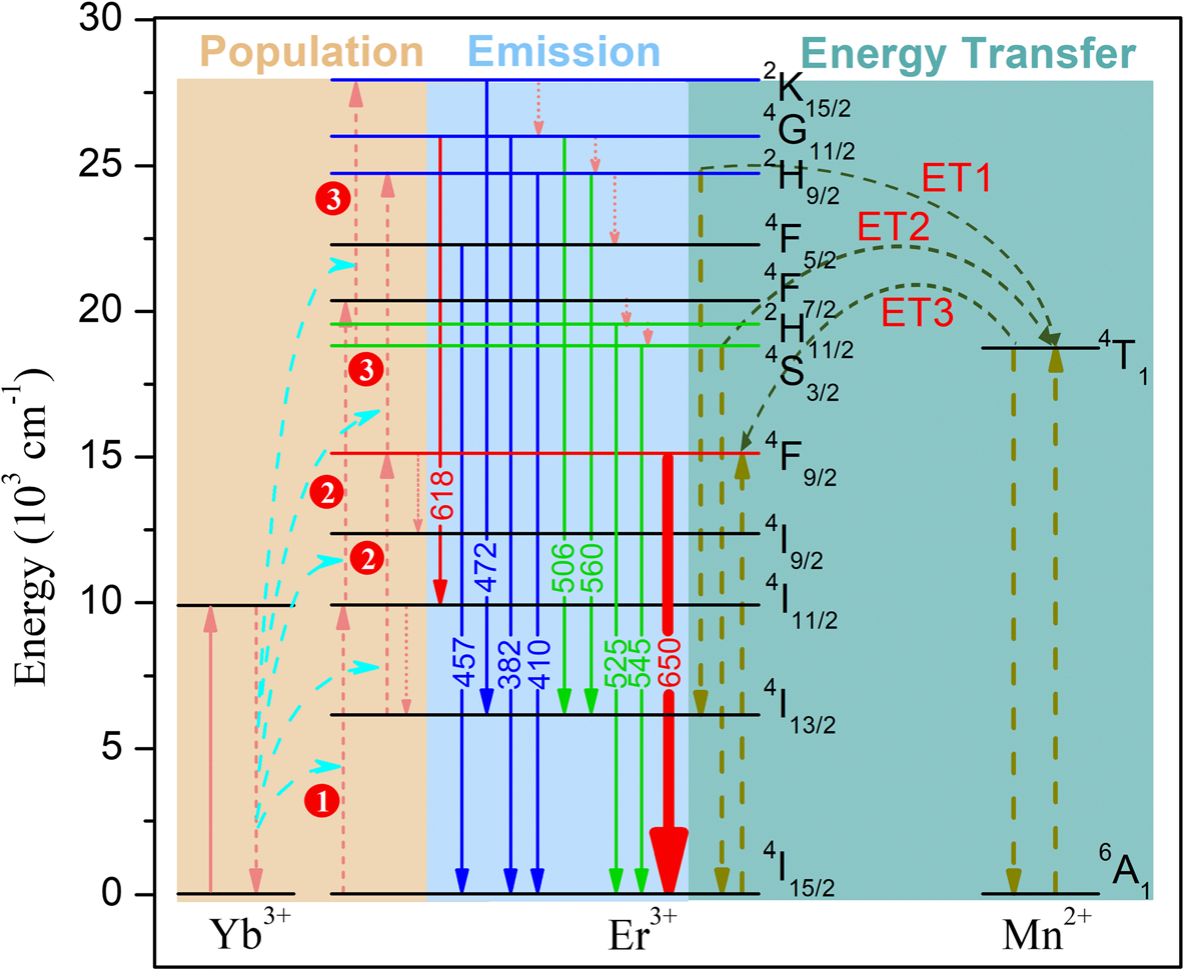
Schematic energy level diagrams for Yb^3+^, Er^3+^, and Mn^2+^ under the 980-nm CW laser excitation, the mechanism of the possible ET, non-radiative transitions, and UC emissions are also presented in the diagram

To further understand the principle of tuning multicolor in the β-NaYF_4_:Yb/Er/Mn microcrystals, we examined the energy level diagram for Yb^3+^, Er^3+^, and Mn^2+^ ions. As shown in Fig. 5, the mechanism of the populating processes, UC emissions, nonradiative transitions, and ET processes are also displayed. For β-NaYF_4_:Yb/Er microcrystals, Yb^3+^ ions absorb the 980 nm incident light, and then populate the Er^3+^ ions from the ground state to the excited states through ET processes. Two possible approaches can promote the higher excited levels of Er^3+^. One is through the green-emitting levels (^4^S_3/2_ and ^2^H_11/2_) populating the ^4^G,^2^K manifold, and the other is through the red-emitting level (^4^F_9/2_) populating the level of ^2^H_9/2_. Once the excited levels of Er^3+^ are populated, significant UC emissions will be generated. Thus, the traditional green (545 nm) and red (650 nm) UC emissions can be easily observed, which exhibit highly efficient UC emissions and have been widely studied. Generally, for 560-nm emission (^2^H_9/2_ → ^4^I_13/2_), the level of ^2^H_9/2_ can be populated through the red-emitting level (^4^F_9/2_), or through the green-emitting levels (^4^S_3/2_, ^2^H_11/2_) populating the ^4^G,^2^K manifold (then followed nonradiative transition to the level of ^2^H_9/2_). Similarly, the 382-, 410-, 457-, 472-, and 506-nm UC emissions are based on the same principle with the population of higher emitting levels of Er^3+^. Moreover, the 618 nm emission originates from populating the ^4^G,^2^K manifold and transition of ^4^G_11/2_ → ^4^I_11/2_.

In addition, for Mn^2+^-doped β-NaYF_4_:Yb/Er microcrystals, the UC luminescence color can be changed from green to red. As shown in Fig. 5, there are two possible ET routes from Er^3+^ to Mn^2+^: one is from the ^2^H_9/2_ → ^4^I_13/2_ transition of Er^3+^ to ^6^A_1_ → ^4^T_1_ transition of Mn^2+^ (process ET1), and another is from the ^4^S_3/2_ → ^4^I_15/2_ transition of Er^3+^ to ^6^A_1_ → ^4^T_1_ transition of Mn^2+^ (process ET2). These two processes (ET1 and ET2) would decrease the 560- and 545-nm UC emissions. When the level ^4^T_1_ of Mn^2+^ is populated, the absorbed energy transfers backward from ^4^T_1_ → ^6^A_1_ transition of Mn^2+^ to ^4^I_15/2_ → ^4^F_9/2_ transition of Er^3+^ (process ET3). This process will promote the population of level ^4^F_9/2_ in Er^3+^ and increase the red (650 nm) UC emission. Therefore, the principle of the tunable color is derived from the nonradiative ET from the levels ^2^H_9/2_ and ^4^S_3/2_ of Er^3+^ to the level ^4^T_1_ of Mn^2+^, then followed by back-ET which increases the population of the level ^4^F_9/2_ in Er^3+^, thereby resulting in the enhancement of R/G ratio [20, 22]. The suppression of traditional green emission (545 nm) and enhancement of red emission signify the strong interaction between Er^3+^ and Mn^2+^ ions, confirming that their ET processes are significant. In the previous studies, the transition of ^2^H_9/2_ → ^4^I_13/2_ was considered as a nonradiative transition approach and rarely emitted at 560-nm UC emission. In fact, as Fig. 5 shows, the 560-nm emission transition is also an ET channel from Er^3+^ to Mn^2+^. Therefore, the 560-nm UC transition will compete with the ET process (ET1) as the pump power increases. Under lower pump power, the absorbed energy mainly populates the lower excited states of Er^3+^ and the new green (560 nm) emission is relatively weaker than the traditional green (545 nm) emission, and simultaneously the process ET1 is insufficient. When the pump power is sufficiently high, the higher excited states of Er^3+^ can be efficiently populated, leading to the competition between the 560-nm emission and process ET1.

**Fig. 6:**
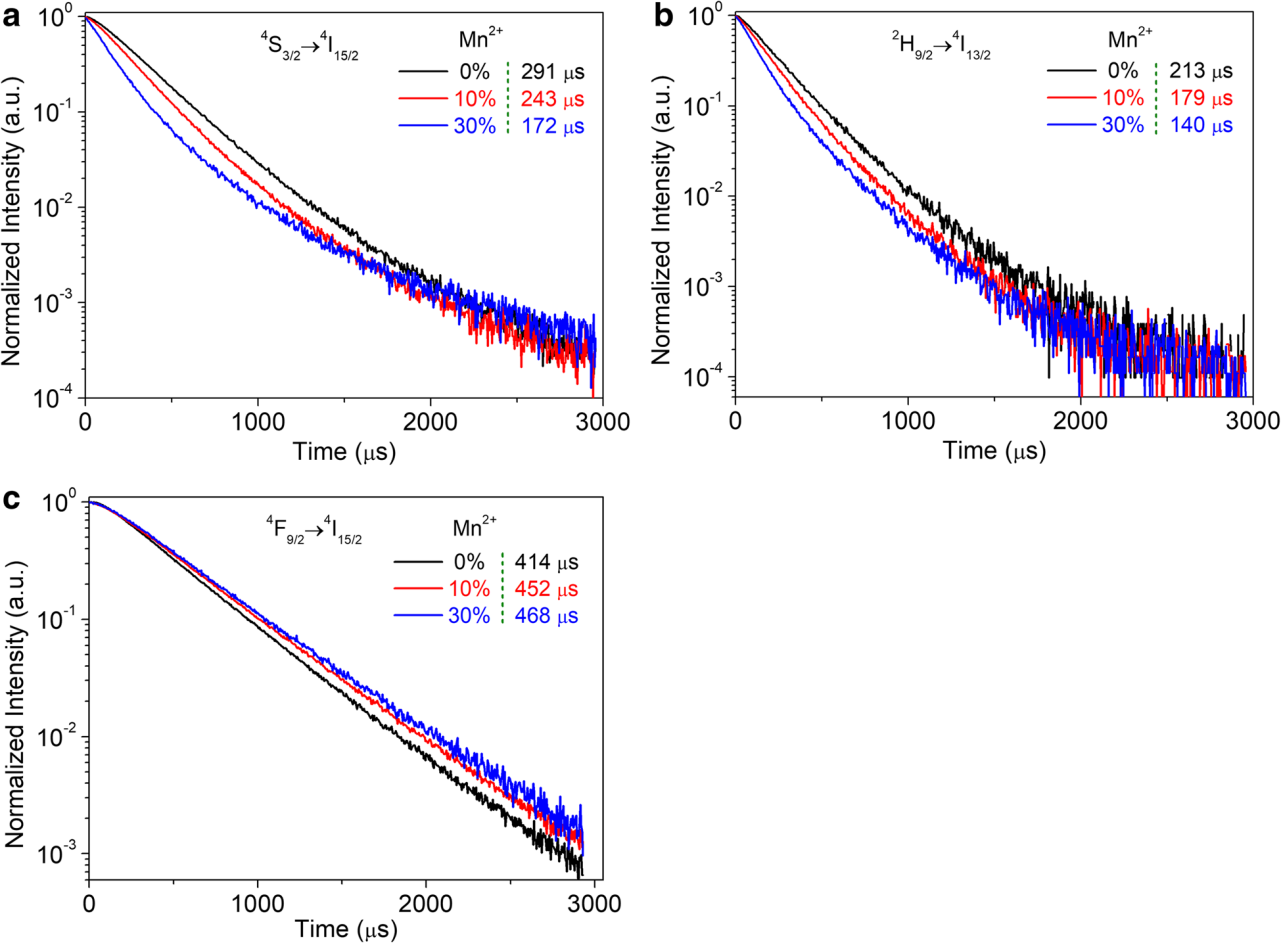
Time-resolved evolutions of the UC emissions from the β-NaYF_4_:Yb/Er (20/2 mol%) microcrystals doping with different amounts of Mn^2+^ ions. **a** (^4^S_3/2_) → ^4^I_15/2_ (545 nm), **b** ^2^H_9/2_ → ^4^I_13/2_ (560 nm), **c** ^4^F_9/2_ → ^4^I_15/2_ (650 nm)

We next examined the time-resolved measurements for the β-NaYF_4_:Yb/Er microcrystals doped with different amounts of Mn^2+^ ions. Figure 6 shows the decay curves and corresponding lifetimes for the red (650 nm), traditional green (545 nm), and new green (560 nm) UC emissions. It can be found that the lifetime of red emission (650 nm) is the longest among the UC emissions. It reveals that the level ^2^H_9/2_ of Er^3+^ can be significantly populated through the red emitting level (^4^F_9/2_). Therefore, we have observed that the 560-nm UC emission becomes more efficient (Figs. 2b and 3a). Notably, the lifetime of the 545- and 560-nm UC emissions tends to decrease as the doping Mn^2+^ ions increase. In contrast, the lifetime of the red (650 nm) emission proposes a declining trend with the doping Mn^2+^ ions increase from 0 to 30 mol%. The reason is that more doping Mn^2+^ ions increase the rate of ET process from Mn^2+^ to Er^3+^, leading to more electrons populating the red emitting level (^4^F_9/2_) of Er^3+^. The conversion efficiency of the processes ET1 and ET2 was obtained using the following equation [19, 42]:

1 $$ \eta =1-\frac{\tau_{\mathrm{Yb}/\mathrm{Er}\left(\mathrm{Mn}\right)}}{\tau_{\mathrm{Yb}/\mathrm{Er}}} $$

where *τ*_Yb/Er(Mn)_ and *τ*_Yb/Er_ represent the lifetime of β-NaYF_4_:Yb/Er microcrystals doping with and without Mn^2+^ ions, respectively. By using the lifetime values from Fig. 6, we can obtain that the efficiency of *η*_1_ was approximately 34% and *η*_2_ was nearly 41% for the β-NaYF_4_:Yb/Er microcrystals doped with 30 mol% Mn^2+^ ions. The results reveal that the processes ET1 and ET2 play an important role in populating the level ^4^T_1_ of Mn^2+^, which leads to an enhancement of the red UC emission based on the process ET3 from Mn^2+^ to Er^3+^. It is worth noting that the *η*_1_ is less than *η*_2_, indicating that the process ET2 is more efficiently than ET1. Therefore, compared with the traditional ratio (650 to 545 nm), the new ratio (650 to 560 nm) remains a lower tunability since the process ET1 is simultaneously acting as radiative transition (560 nm UC emission) and an ET channel from Er^3+^ to Mn^2+^.

## Conclusion

In conclusion, we have demonstrated the tunable multicolor display from a single Mn^2+^-doped β-NaYF_4_:Yb/Er microcrystal by individual optical characterization. The tuning multicolor from green to red was realized in a single β-NaYF_4_:Yb/Er microcrystal by varying the doping amounts of Mn^2+^ ions. Under high power excitation, the newly emerged green (560 nm) UC emission modifies the region of tuning R/G ratio. Further investigations suggest that the tunable multicolor not only depends on the doping concentrations of Mn^2+^ ions, but also relies on the excitation intensities. Our work presents a new approach for understanding the tunable multicolor in Mn^2+^-doped with Yb^3+^/Er^3+^ microcrystals. We believe that the tunable color for the single microcrystal provides potential prospects in both color display and micro-optoelectronic devices.

## Abbreviations

CCD: Charge-coupled device
CW: Continuous Wave
DI: Deionized
ET: Energy transfer
Mn^2+^: Manganese ions
NIR: Near-infrared
R/G: Red-to-green
SEM: Scanning electron microscopy
UC: Upconversion
XRD: X-ray diffraction

## References

[1] Wang Y, Zheng K, Song S, Fan D, Zhang H, Liu X (2018). Remote manipulation of upconversion luminescence. Chem Soc Rev.

[2] Ma Y, Yang Z, Zhang H, Qiu J, Song Z (2018). Preparation, growth mechanism, upconversion, and near-infrared photoluminescence properties of convex-lens-like NaYF_4_ microcrystals doped with various rare earth ions excited at 808 nm. Cryst Growth Des.

[3] Chen B, Kong W, Liu Y, Lu Y, Li M, Qiao X, Fan X, Wang F (2017). Crystalline hollow microrods for site-selective enhancement of nonlinear photoluminescence. Angew Chem Int Ed.

[4] Wang F, Han Y, Lim CS, Lu Y, Wang J, Xu J, Chen H, Zhang C, Hong M, Liu X (2010). Simultaneous phase and size control of upconversion nanocrystals through lanthanide doping. Nature.

[5] Liu Y, Lu Y, Yang X, Zheng X, Wen S, Wang F, Vidal X, Zhao J, Liu D, Zhou Z, Ma C, Zhou J, Piper JA, Xi P, Jin D (2017). Amplified stimulated emission in upconversion nanoparticles for super-resolution nanoscopy. Nature.

[6] Zhan Q, Liu H, Wang B, Wu Q, Pu R, Zhou C, Huang B, Peng X, Ågren H, He S (2017). Achieving high-efficiency emission depletion nanoscopy by employing cross relaxation in upconversion nanoparticles. Nat Commun.

[7] Meruga JM, Baride A, Cross W, Kellara JJ, May PS (2014). Red-green-blue printing using luminescence-upconversion inks. J Mater Chem C.

[8] You M, Zhong J, Hong Y, Duan Z, Lin M, Xu F (2015). Inkjet printing of upconversion nanoparticles for anti-counterfeit applications. Nanoscale.

[9] Bian W, Lin Y, Wang T, Yu X, Qiu J, Zhou M, Luo H, Yu SF, Xu X (2018). Direct identification of surface defects and their influence on the optical characteristics of upconversion nanoparticles. ACS Nano.

[10] Fernandez-Bravo A, Yao K, Barnard ES, Borys NJ, Levy ES, Tian B, Tajon CA, Moretti L, Altoe MV, Aloni S, Beketayev K, Scotognella F, Cohen BE, Chan EM, Schuck PJ (2018). Continuous-wave upconverting nanoparticle microlasers. Nat Nanotechnol.

[11] Wang T, Yu H, Siu CK, Qiu J, Xu X, Yu SF (2017). White-light whispering-gallery-mode lasing from lanthanide doped upconversion NaYF_4_ hexagonal microrods. ACS Photonics.

[12] Damasco JA, Chen G, Shao W, Ågren H, Huang H, Song W, Lovell JF, Prasad PN (2014). Size-tunable and monodisperse Tm^3+^/Gd^3+^-doped hexagonal NaYbF_4_ nanoparticles with engineered efficient near infrared-to-near infrared upconversion for in vivo imaging. ACS Appl Mater Interfaces.

[13] Labrador-Páez L, Ximendes EC, Rodríguez-Sevilla P, Ortgies DH, Rocha U, Jacinto C, Rodríguez EM, Haro-González P, Jaque D (2018). Core–shell rare-earth-doped nanostructures in biomedicine. Nanoscale.

[14] Kang N, Liu Y, Zhou Y, Wang D, Chen C, Ye S, Nie L, Ren L (2016). Phase and size control of core–shell upconversion nanocrystals light up deep dual luminescence imaging and CT in vivo. Adv Healthc Mater.

[15] Auzel F (2014). Upconversion and anti-stokes processes with f and d ions in solids. Chem Rev.

[16] Chan EM, Han G, Goldberg JD, Gargas DJ, Ostrowski AD, Schuck PJ, Cohen BE, Milliro DJ (2012). Combinatorial discovery of lanthanide-doped nanocrystals with spectrally pure upconverted emission. Nano Lett.

[17] Chen D, Liu L, Huang P, Ding M, Zhong J, Ji Z (2015). Nd^3+^-sensitized Ho^3+^ single-band red upconversion luminescence in core−shell nanoarchitecture. J Phys Chem Lett.

[18] Wang F, Liu XG (2008). Upconversion multicolor fine-tuning: visible to near-infrared emission from lanthanide-doped NaYF_4_ nanoparticles. J Am Chem Soc.

[19] Chen G, Liu HC, Somesfalean G, Liang HJ, Zhang ZG (2009). Upconversion emission tuning from green to red in Yb^3+^/Ho^3+^ codoped NaYF_4_ nanocrystals by tridoping with Ce^3+^ ions. Nanotechnology.

[20] Wang J, Wang F, Wang C, Liu Z, Liu XG (2011). Single-band upconversion emission in lanthanide-doped KMnF_3_ nanocrystals. Angew Chem Int Ed.

[21] Wu M, Song EH, Chen ZT, Ding S, Ye S, Zhou JJ, Xu SQ, Zhang QY (2016). Single-band red upconversion luminescence of Yb^3+^–Er^3+^ via nonequivalent substitution in perovskite KMgF_3_ nanocrystals. J Mater Chem C.

[22] Tian G, Gu Z, Zhou L, Yin W, Liu X, Yan L, Jin S, Ren W, Xing G, Li S, Zhao Y (2012). Mn^2+^ dopant-controlled synthesis of NaYF_4_:Yb/Er upconversion nanoparticles for in vivo imaging and drug delivery. Adv Mater.

[23] Xie MY, Peng XN, Fu XF, Zhang JJ, Li GL, Yu XF (2009). Synthesis of Yb^3+^/Er^3+^ co-doped MnF_2_ nanocrystals with bright red up-converted fluorescence. Scripta Mater.

[24] Zhang Y, Wang F, Lang Y, Yin J, Zhang M, Liu X, Zhang D, Zhao D, Qin G, Qin W (2015). KMnF_3_:Yb^3+^,Er^3+^@KMnF_3_:Yb^3+^ active-core–active-shell nanoparticles with enhanced red upconversion fluorescence for polymer-based waveguide amplifiers operating at 650 nm. J Mater Chem C.

[25] Liu T, Li S, Liu Y, Guo Q, Wang L, Liu D, Zhou J (2016). Mn-complex modified NaDyF_4_:Yb@NaLuF_4_:Yb,Er@polydopamine core–shell nanocomposites for multifunctional imaging-guided photothermal therapy. J Mater Chem B.

[26] Lu H, Hao H, Zhu H, Shi G, Fan Q, Song Y, Wang Y, Zhang X (2017). Enhancing temperature sensing behavior of NaLuF_4_:Yb^3+^/Er^3+^ via incorporation of Mn^2+^ ions resulting in a closed energy transfer. J Alloy Compd.

[27] Zhou Y, Ling B, Chen H, Wang L (2018). Mn^2+^-doped NaYF_4_:Yb,Er upconversion nanoparticles for detection of uric acid based on the Fenton reaction. Talanta.

[28] Guo X, Wu S, Duan N, Wang Z (2016). Mn^2+^-doped NaYF_4_:Yb/Er upconversion nanoparticle-based electrochemiluminescent aptasensor for bisphenol A. Anal Bioanal Chem.

[29] Liu M, Ye Y, Yao C, Zhao W, Huang X (2014). Mn^2+^-doped NaYF_4_:Yb/Er upconversion nanoparticles with amplified electrogenerated chemiluminescence for tumor biomarker detection. J Mater Chem B.

[30] Li C, Zhang C, Hou Z, Wang L, Quan Z, Lian H, Lin J (2009). β-NaYF_4_ and β-NaYF_4_:Eu^3+^ microstructures: morphology control and tunable luminescence properties. J Phys Chem C.

[31] Chen B, Sun T, Qiao X, Fan X, Wang F (2015). Directional light emission in a single NaYF_4_ microcrystal via photon upconversion. Adv Opt Mater.

[32] Gao D, Zhang X, Chong B, Xiao G, Tian D (2017). Simultaneous spectra and dynamics processes tuning of a single upconversion microtube through Yb^3+^ doping concentration and excitation power. Phys Chem Chem Phys.

[33] Gao W, Kong X, Han Q, Dong J, Zhang W, Zhang B, Yan X, Zhang Z, He E, Zheng H (2018). Highly efficient multi-colour upconversion emission of Yb^3+^/Er^3+^, Ho^3+^ codoped single LiYF_4_ octahedral microparticle. J Lumin.

[34] Wang F, Deng R, Liu X (2014). Preparation of core-shell NaGdF_4_ nanoparticles doped with luminescent lanthanide ions to be used as upconversion-based probes. Nat Protoc.

[35] Yuan M, Wang R, Zhang C, Yang Z, Cui W, Yang X, Xiao N, Wang H, Xu X (2018). Exploiting the silent upconversion emissions from a single β-NaYF_4_:Yb/Er microcrystal via saturated excitation. J Mater Chem C.

[36] Shannon RD (1976). Revised effective ionic radii and systematic studies of interatomie distances in halides and chaleogenides. Acta Cryst A.

[37] Tian D, Gao D, Chong B, Liu X (2015). Upconversion improvement by the reduction of Na^+^-vacancies in Mn^2+^ doped hexagonal NaYbF_4_:Er^3+^ nanoparticles. Dalton Trans.

[38] Pollnau M, Gamelin DR, Lüthi SR, Güdel HU, Hehlen MP (2000). Power dependence of upconversion luminescence in lanthanide and transition-metal-ion systems. Phys Rev B.

[39] Suyver JF, Aebischer A, García-Revilla S, Gerner P, Güdel HU (2005). Anomalous power dependence of sensitized upconversion luminescence. Phys Rev B.

[40] Zhou J, Chen G, Zhu Y, Huo L, Mao W, Zou D, Sun X, Wu E, Zeng H, Zhang J, Zhang L, Qiu J, Xu S (2014). Intense multiphoton upconversion of Yb^3+^–Tm^3+^ doped β-NaYF_4_ individual nanocrystals by saturation excitation. J Mater Chem C.

[41] Kaiser M, Würth C, Kraft M, Hyppänen I, Soukka T, Resch-Genger U (2017) Power-dependent upconversion quantum yield of NaYF_4_:Yb^3+^,Er^3+^ nano- and micrometer-sized particles–measurements and simulations. Nanoscale 9:10051–10058 10.1039/c7nr02449e 28686275

[42] De la Rosa E, Salas P, Desirena H, Angeles C, Rodríguez RA (2005). Strong green upconversion emission in ZrO_2_ :Yb^3+^–Ho^3+^ nanocrystals. Appl Phys Lett.

